# Diacylated 1,10‐diaza‐18‐crown‐6 as an Alternative Template for Fluorescent Sensors of Calcium Ions

**DOI:** 10.1002/ansa.202400034

**Published:** 2024-11-03

**Authors:** Oscar G. Smith, Jennifer C. Anene, Simon Wheeler

**Affiliations:** ^1^ Leicester School of Pharmacy De Montfort University The Gateway, Leicester UK

## Abstract

The performance of many known fluorescent sensors for Ca^2+^ is pH‐dependent. We set out to address this by preparing a novel calcium sensor based on diacylated 1,10‐diaza‐18‐crown‐6 and containing no basic centres. The sensor is sensitive to sub‐millimolar levels of Ca^2+^ and partially tolerant of an aqueous environment. However, it suffers from a lack of selectivity over some other divalent cations, and retained pH sensitivity in the acidic range. The relations between these properties and the sensor's structure are discussed.

**Abbreviations**
HEPES: (4‐(2‐hydroxyethyl)‐1‐piperazineethanesulfonic acid)
ICT: intramolecular charge transfer
MeCN: acetonitrile
MeOH: methanol
PET: photo‐induced electron transfer

## 1 Introduction

While calcium indicator Fura‐2 and its intellectual successors remain invaluable tools for intracellular calcium imaging [1, 2] their reliance on ionised carboxylates and basic nitrogens for metal ion coordination renders their use in low pH environments problematic. Crown ethers represent a potential way of avoiding this problem by offering a cation‐binding site which can be polar but uncharged across the pH range. Several N‐aryl aza‐crown‐based sensors have been reported on the principle that Ca^2+^ complexation by the crown disrupts intramolecular charge transfer (ICT) from the crown nitrogen to a conjugated fluorophore thereby altering fluorescence. It appears, however, that we lack a fundamental understanding of such molecules as there are examples where fluorescence decreases [3, 4] as well as those where it increases, eg [5]. More relevant to this discussion are the repeated reports [6, 7, 8] that the emission from such sensors is affected by H^+^ rendering them pH‐sensitive (cf [9] where fluorescence from a structurally similar sensor increases below pH 5). Photo‐induced electron transfer (PET) is a complementary phenomenon to ICT as electron transfer occurs through space to quench the excited state of a fluorophore. Multiple examples exist of the exploitation of this process as the basis of sensors for metal ions which feature crown ethers as the metal binding unit (reviewed [10]). Co‐ordination of metal ions by the crown reduces the availability of lone pairs and blocks the PET process resulting in an increase in fluorescence. Outstanding applications of this idea to fluorescent calcium sensors are molecules whose emission increases by over 100x on the addition of Ca^2+^ [11, 12, 13], though all three sensors feature N‐aryl aza‐crowns so it is likely that response is pH‐dependent (this was shown in one case [12]). Another of these studies [13] found that the addition of even small amounts of water significantly reduced the fluorescent response, a result echoed by studies on a related polyether Ca^2+^ sensor [14]. More generally the interactions of crown ethers with associated waters form an ongoing area of research [15, 16, 17] into the subtleties of this class of molecules which are still incompletely understood almost 60 years after their discovery.

**FIGURE 1:**
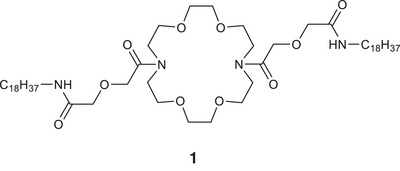
Diacylated crown ether previously used as the basis for an electrochemical calcium sensor.

Multiple studies in the application of crown ethers to calcium‐sensing have demonstrated the coordination of carbonyl oxygen to the metal ion [18, 19, 20, 21, 22, 23, 24] which offers the possibility of designing a metal binding site with 3‐dimensional character (for early studies see [25]). This may explain the effectiveness of the work from Koji Suzuki's lab [26] which reported electrochemical sensors based on diacylated diaza‐crowns, eg **1** (Figure 1), embedded in a polymer membrane (compare [27]). The sensors displayed remarkable sensitivity for Ca^2+^ (detection of 5 × 10^−5^ M) and selectivity (up to 10^5^) over related ions. A more recent density functional theory study [28] has supported the idea that such compounds would use both carbonyl oxygens to coordinate the metal ion. We reasoned that a diacylated diaza‐crown would serve as an alternative template for a fluorescent sensor—the ion binding properties would be retained but incorporated fluorophores would give a fluorescent response on calcium binding via blocking PET. The large Stokes’ shift and easy synthetic modification of the naphthalimide moiety have led to its widespread use in fluorescent sensors (reviewed [29]) thus we adopted that as our fluorophore and proposed structure **5** as a fluorescent calcium sensor. We report here our preparation of this molecule and our initial explorations of its properties.

## 2 Results and Discussion

**SCHEME 1:**
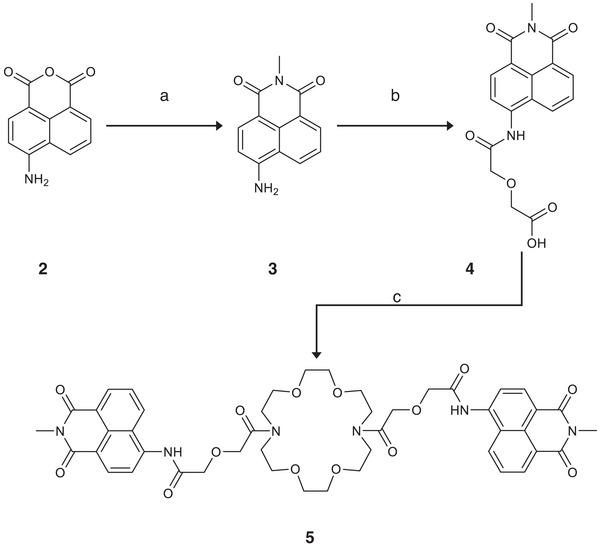
Synthesis of sensor **5** reagents and conditions: (a) MeNH_2_, EtOH/H_2_O, reflux, 24 h, quantitative; (b) diglyocolic anhydride, py, reflux, 7 days, 41%; (c) 1,10‐diaza‐18‐crown‐6, *N,N*‐bis(2‐oxo‐3‐oxazolidinyl)]phosphinic chloride Et_3_N, DCM, 20%.

Sensor **5** was prepared as shown in Scheme 1. Known [30] anhydride **2** was treated with an excess of methylamine to yield naphthalimide **3**. The linker was then installed by using the amino group to perform a ring‐opening reaction on diglycolic anhydride to yield acid **4**. Coupling with 1,10‐diaza‐18‐crown‐6 under previously described conditions [26] yielded our desired sensor **5**. The photophysical characteristics (λ_ex_, λ_em_, ε) of **3** and **4** were broadly in line with previously disclosed analogues [31, 32, 33] but the extinction coefficient of **5** (2300 M^−1^ cm^−1^, Figure S1) was rather lower than that of previous compounds [31, 33] and **4**. Absorption and emission wavelengths of **5** (λ_ex_ = 351 nm, λ_em_ = 462 nm) were very similar to those previously published for 4‐amidonaphthalimides [31, 32, 33]. We speculate that this drop in ε may be due to a ground‐state interaction between the bis‐amido crown and the naphthalimide.

**FIGURE 2:**
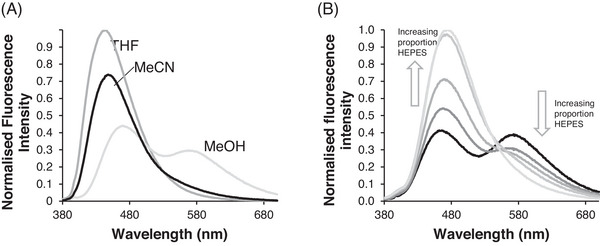
Excimer properties of sensor **5**: (A) Emission spectrum in various solvents. (B) Increasing the proportion of (4‐(2‐hydroxyethyl)‐1‐piperazineethanesulfonic acid) (HEPES) (10 mM, pH 7.4) increases emission from monomer and decreases emission from excimer. For all experiments [5] = 10 µM, λ_ex_ = 355 nm.

While the fluorescence spectra of **5** in acetonitrile (MeCN) and tetrahydrofuran gave single peaks the spectrum in methanol (MeOH) had an additional peak at a longer wavelength (Figure 2A) which we assigned to excimer emission. We initially attributed this to the polarity of the medium [34, 35] but experiments with mixtures of MeCN and (4‐(2‐hydroxyethyl)‐1‐piperazineethanesulfonic acid) (HEPES) buffer (10 mM, pH 7.4) problematised this. Excimer emission (575 nm) was pronounced with 20% HEPES but monomer emission (462 nm) then increased relative to excimer intensity (Figure 2B) in a linear fashion (Figure S2A) with higher proportions. Absorption was unaffected by variations in the proportion of HEPES (Figures 2B, while excitation spectra for monomer and excimer were almost identical as expected (Figure S2C). Although we do not yet understand why excimer should appear at a low proportion of HEPES and then disappear again at higher proportions it is noteworthy that exactly similar results were obtained when using phosphate‐buffered saline as a buffer in place of HEPES (Figure S2D,E) and that other such sharp discontinuities in fluorescence of naphthalimides in partially aqueous media have been observed previously [32, 36]. Tentatively we attribute the appearance of the excimer peak to an aggregation effect (see [37] for a previous example of this with a naphthalimide) as monomer intensity increases relative to excimer intensity at lower concentrations in both 20% HEPES/MeCN and in MeOH (Figure S2F,G).

**FIGURE 3:**
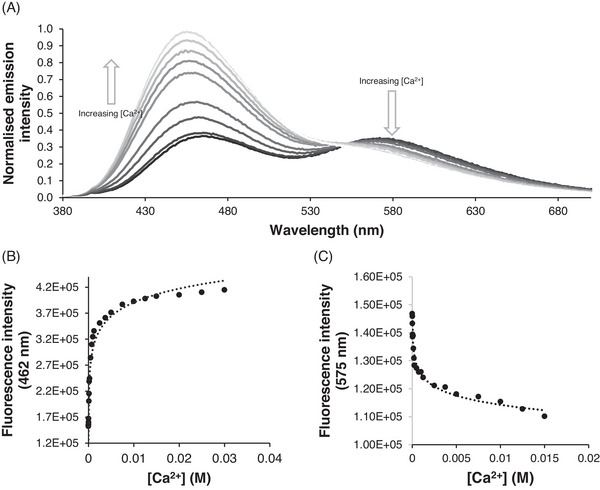
Fluorescence changes on the addition of Ca^2+^ to sensor **5**: (A) Addition of increasing concentrations of Ca^2+^ leads to an increase in monomer and a decrease of excimer. (B) Titration experiment at monomer wavelength. (C) Titration experiment at excimer wavelength. All experiments were conducted in 20% (4‐(2‐hydroxyethyl)‐1‐piperazineethanesulfonic acid) (HEPES)/acetonitrile (MeCN), [5] = 10 µM, λ_ex_ = 355 nm. Curves are simple logarithmic fits.

We proceeded to examine the effects of Ca^2+^ on emission from sensor **5**. In 20% HEPES (10 mM, pH 7.4)/MeCN, we were pleased to find that monomer emission increased gradually to a maximum of ca. 2.7‐fold higher than the sensor alone (Figure 3A). We attribute this change to Ca^2+^ binding inside the crown ether ring blocking PET fluorescence quenching and so giving emission enhancement. This interpretation is supported by the observation of identical fluorescence enhancements with three different calcium salts (Figure S3A) cf [38, 39]. However, we also observed small absorbance changes on adding Ca^2+^ (Figure S3B,C) which may imply a Ca^2+^‐induced disruption of the postulated ground‐state interaction between bis‐amido crown ether and napthalimide and may in turn suggest that our sensor does not work by a pure PET mechanism. [40] We also observed a decline in excimer emission (Figure 3A) which followed a biphasic profile (Figure 3C). We hypothesise that this reveals two binding events. The first is the binding of Ca^2+^ with high affinity inside the crown giving a cationic complex, thus reducing aggregation and thereby excimer emission. The second, low affinity, binding event of Ca^2+^ is likely to the naphthalimide or the linker. This might also explain the gradual blue shift of the monomer peak (Figure 3A). Consistent with this interpretation we were able to fit the data (Figure 3B) to a 1:2 binding model using BindFit (http://supramolecular.org) [40] and obtained log *K*_11_ = 3.4, log *K*_12_ = 1.5. The equivalent experiment in 40% HEPES (10 mM, pH 7.4) / MeCN gave a smaller fluorescence enhancement (Figure S4A) resulting from less potent binding (log *K*_11_ = 2.8, log *K*_12_ = 1.7; Figure S4B). Further increases in the proportion of aqueous components gave even lower emission enhancements (Figure S4C). A higher proportion of aqueous components renders less favourable the necessary dehydration of both Ca^2+^ and crown ether before ion binding can occur, which we hypothesise can rationalise these observations. Whilst this performance is short of optimal the ability of our sensor to sense calcium at all in a 40% aqueous system marks it out as significantly more water tolerant than any other fluorescent Ca^2+^ sensor using unionised groups.

**FIGURE 4:**
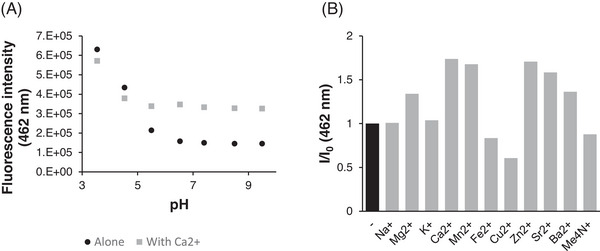
Effect of other species on emission from sensor **5**: (A) Emission from **5** (10 µM) is invariant (both alone and in the presence of 5 mM Ca^2+^) above pH 6.5 but increases at more acidic values. (B) Emission enhancement from **5** in the presence of 1 mM various cations. (Cations were added as perchlorate salts as solutions in acetonitrile (MeCN) with the exception of Me_4_NClO_4_ which was added as a solution in dimethyl sulfoxide (DMSO). All experiments were conducted in 20% (4‐(2‐hydroxyethyl)‐1‐piperazineethanesulfonic acid) (HEPES) (10 mM, pH 7.4)/MeCN, [5] = 10 µM, λ_ex_ = 355 nm.

We next asked if emission from the sensor was pH‐independent, both alone and in the presence of Ca^2+^. Although fluorescence did not vary with pH in the range of 6.5–9.5 emission from the sensor alone increased significantly at more acidic values and the addition of Ca^2+^ did not further enhance it (Figure 4A). We postulate that this arises not from protonation of our sensor per se but from its ability to form H‐bonds with H_3_O^+^, which would also explain why our sensor shows reduced responses in more aqueous media (Figure S4). To our knowledge H‐bonding of H_3_O^+^ has not been systematically studied with aza‐crowns but has been extensively investigated with standard crown ethers [41, 42]. The use of lone pairs on the ring oxygens in this manner reduces the PET quench of naphthalimide emission thereby rendering **5** insensitive to calcium ions. To investigate the selectivity of our sensor we added 1 mM metal perchlorate salts to 10 µM sensor in 20% HEPES/MeCN. We were pleased to discover that our sensor did not respond at all to monovalent cations (Figure 4B and Figure S5A) which we attribute to the preference of relatively hard amide donors for ions with higher charge densities. We also observed virtually no change in emission of 10 µM sensor even with 100 mM Na^+^ or K^+^ (Figure S5B). **5** showed only limited selectivity for Ca^2+^ over other group II ions (Figure 4B and Figure S5C) and over other dications in general (Figure 4B and Figure S5D). In particular, our sensor gave an emission change with Zn^2+^ comparable to that observed with Ca^2+^ (Figure 4B and Figures S5D and S6A). This is likely driven by similar binding constants (log *K*_11_ = 4.0, log *K*_12_ = 1.9; Figure S6B), though changes in the form of both the absorbance and emission spectra (Figure S6A,C) suggest that this may derive at least partially from direct interaction between Zn^2+^ and the napthalimide. The general lack of selectivity towards other divalent cations likely arises from the relative flexibility of our molecule and in particular the ability of the carbonyls to adopt a range of O ^…^ M^2+^ distances. The exceptions to this pattern were Cu^2+^ and Fe^2+^ which caused appreciable fluorescence quenching (Figure 4B and Figure S5E) presumably through a paramagnetic mechanism (cf [32]).

## 3 Conclusion

In summary, we have presented an example of an alternative template for the design of fluorescent sensors of Ca^2+^. This compound shows interesting aggregation‐driven behaviour in partially aqueous media, excellent selectivity over monovalent cations and a greater water tolerance than previously disclosed related sensors. Future work will focus on improving selectivity over divalent cations and making a sensor that is functional in aqueous environments for potential biological applications. These investigations are underway in our laboratory and will be reported in due course.

## Conflicts of Interest

The authors declare no conflicts of interest.

## Supporting information



Supporting Information

## Data Availability

The data that support the findings of this study are available from the corresponding author upon reasonable request.
