# Supplementary material for: Diacylated 1,10‐diaza‐18‐crown‐6 as an Alternative Template for Fluorescent Sensors of Calcium Ions
Source: Anal Sci Adv. 2024 Nov 3;5(11-12):e202400034. doi: 10.1002/ansa.202400034 (PMC11627183; doi:10.1002/ansa.202400034)
Supplement: Supplementary file 1 — Supporting Information [file ANSA-5-e202400034-s001.docx]

Supporting Information for

**Diacylated 1,10-diaza-18-crown-6 as an alternative template for fluorescent sensors of calcium ions**

Oscar G. Smith, Jennifer C. Anene and Simon Wheeler (0000-0003-0215-8648)

Leicester School of Pharmacy, De Montfort University, The Gateway, Leicester, LE1 9BH, UK

**Synthesis**

Chemicals were obtained from commercial suppliers and used without further purification. NMR spectroscopy was carried out in the stated deuterated solvents using a JEOL 600 MHz spectrometer at 298 K. Chemical shifts are expressed in ppm.

Preparation of **3**

To a stirred suspension of anhydride xx prepared as previously described^1^ (110 mg, 0.52 mmol, 1 equiv) in EtOH (2 mL) was added MeNH_2_ (40 w/w% sol^n^ in water, 161 μL, 2.07 mmol, 4 equiv) and the whole heated at reflux for 24 hours. Solvent was removed in vacuo to yield product as an orange-brown solid (117 mg, 0.52 mmol, 100%).

^1^H NMR (DMSO-*d*_6_, 600MHz): 3.34 (3H, s, H11), 6.84 (1H, d, J=8.4Hz, H3), 7.40 (2H, bs, H12), 7.63 (1H, appears t, J=7.6Hz, H6), 8.18 (1H, d, J=8.1Hz, H2), 8.41 (1H, d, J=7.2Hz, H7), 8.59 (1H, d, J=8.3Hz, H5)

^13^C NMR (DMSO-*d*_6_, 100MHz): 26.9 (C11), 108.2 (C1), 108.7 (C3), 119.9 (C4a), 122.3 (C8), 124.5 (C6), 129.8 (C5), 130.1 (C8a), 131.4 (C7), 134.4 (C2), 153.2 (C4), 163.7 (C10), 164.6 (C9)

UV-Vis (MeCN): λ_ex_ = 258, 413 nm; λ_em_ = 519 nm; ε = 6200 M^-1^ cm^-1^

Preparation of **4**

To a stirred suspension of amine **3** (100 mg, 0.44 mmol, 1 equiv) in pyridine (2 mL) was added diglycolic anhydride (103 mg, 0.89 mmol, 2 equiv) and the whole heated at 110°C in a ReactiVial for 7 days. Solvent was removed in vacuo and the residue purified by column chromatography over silica eluting with 50 – 100% EtOAc/DCM. Relevant fractions were combined and evaporated to yield product as a yellow solid containing some residual diglycolic anhydride (63 mg, 0.18 mmol, 41%).

^1^H NMR (DMSO-*d*_6_, 600MHz): 3.35 (3H, s, H11), 4.27 (2H, s, H15), 4.37 (2H, s, H14), 7.84 (1H, appears t, J=7.6 Hz, H6), 8.25 (1H, d, J=8.1 Hz, H3), 8.45 (1H, d, J=8.1 Hz, H2), 8.48 (1H, d, J=7.1 Hz, H7), 8.56 (1H, d, J=8.4 Hz, H5), 10.41 (1H, s, H12)

^13^C NMR (DMSO-*d*_6_, 100MHz): 27.2 (C11), 68.8 (C15), 71.1 (C14), 118.7 (C1), 120.3 (C3), 122.9 (C8), 124.9 (C4a), 127.1 (C6), 128.7 (C8a), 129.4 (C5), 131.3 (C7), 131.9 (C2) 139.9 (C4), 163.7 (C10), 164.3 (C9), 169.7 (C13), 172.6 (C16)

UV-Vis (MeCN): λ_ex_ = 363 nm; λ_em_ = 454 nm. ε ≈ 34,000 Low solubility prevented more accurate determination of ε.

Preparation of **5**

To a stirred solution of acid **4** (23 mg, 0.07 mmol, 1 equiv) in DCM (5 mL) was added Et_3_N (18.7 μL, 0.13 mmol, 2 equiv) followed by N,N-bis(2-oxo-3-oxazolidinyl)]phosphinic chloride (18.3 mg, 0.07 mmol, 1 equiv) and finally 1,10-diaza-18-crown-6 (9.2 mg, 0.035 mmol, 0.5 equiv). The whole was stirred for 16 hours at room temperature. Solvent was removed in vacuo and the residue loaded directly onto a column of silica eluting with EtOAc, then with 10% MeOH/DCM and finally with 90:10:1 DCM/MeOH/880 NH_3_. Relevant fractions were combined and evaporated to yield product as an amorphous yellow solid (6 mg, 0.007 mmol, 20%).

^1^H NMR (D_2_O, 600MHz): 3.22 (6H, s, H11), 3.36 (12H, m, H17-19), 3.83 (12H, m, H17-19), 4.27 (4H, s, H15), 4.45 (4H, s, H14), 7.52 (1H, appears t, J=7.8 Hz, H6), 7.78 (1H, d, J=7.9 Hz, H3), 7.92 (1H, d, J=7.9 Hz, H2), 8.04 (2H, m, H5, 7).

^13^C NMR (D_2_O, 100MHz): 26.7 (C11), 47.4 (C19), 65.4 (C18), 69.6 (C17), 70.4 (C14), 70.7 (br, C15), 117.5 (C1), 120.58 (C3), 120.62 (C8), 124.0 (C4a), 126.9 (C6), 127.2 (C8a), 128.9 (C5), 131.6 (C7), 131.7 (C2), 138.3 (C4), 164.5 (C10), 165.2 (C9), 171.8 (C13), 177.1 (C16).

HRMS: mass ion fragments into diazacrown and side chains. For diazacrown C_12_H_26_N_2_O_4_.H^+^ requires 263.1971, found 263.1963; C_12_H_26_N_2_O_4_.2H^2+^ requires 132.1025, found 132.1019, C_12_H_26_N_2_O_4_.Na^+^ requires 285.1790, found 285.1782. For acid side chain C_17_H_14_N_2_O_6_.H^+^ requires 343.0930, found 343.1840

UV-Vis (MeCN): λ_ex_ = 351 nm; λ_em_ = 448 nm; ε = 2300 M^-1^ cm^-1^


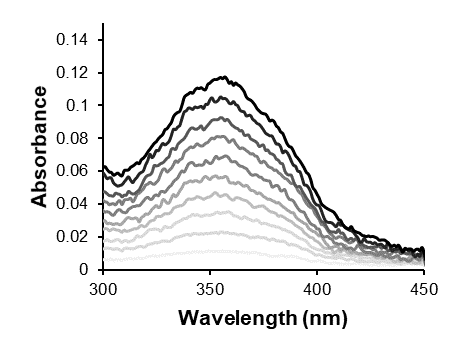

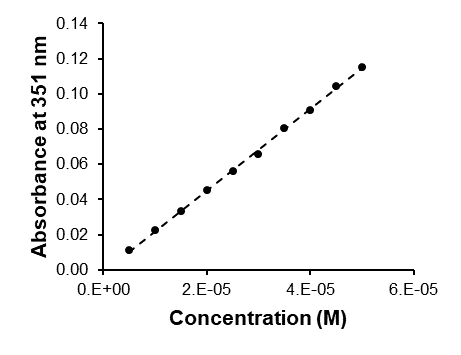


**Figure S1 Absorbance properties of sensor 5 in MeCN A** Absorbance spectra **B** Determination of ε (= 2300, r^2^ = 0.999)

**A**

**B**

**Fluorescence experiments**

Photophysical experiments were conducted in a quartz cuvette using an Edinburgh Instruments FS5 fluorimeter with Fluoracle software. Step size was 1 nm and dwell time was 0.2 s; excitation and emission slit widths were 1.6 nm. All experiments used λ_ex_ = 355 nm and recorded emission in the range 380 – 700 nm. HEPES refers to a 10 mM solution adjusted to pH 7.4 unless otherwise stated. Solvents were standard grade and stored over activated 4 Å sieves. Sensor **5** was stored as a 1 mM solution in MeCN at 4 °C and diluted as necessary. Cations were stored as 200 mM solutions of perchlorate salts in MeCN unless otherwise stated. Binding titration data were fitted using BindFit (<http://supramolecular.org>) ^2^ ; the fits for [20% HEPES](http://app.supramolecular.org/bindfit/view/d39aac90-73f7-4dd1-a84a-443d136c3d7d) and [40% HEPES](http://app.supramolecular.org/bindfit/view/4a82cbf6-150a-4bd5-b865-57095b3ae4d4) are publicly available.


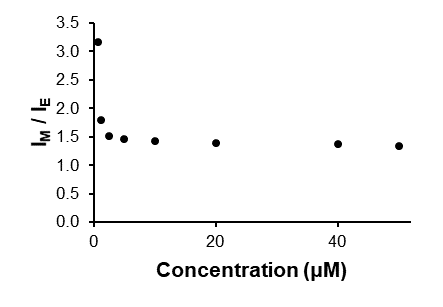

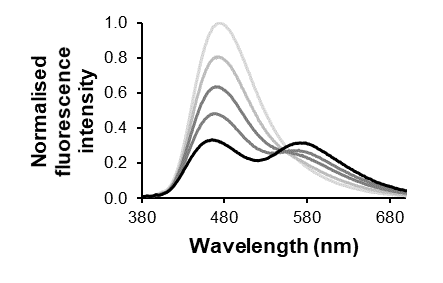

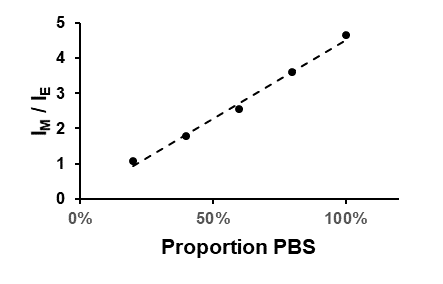


**Figure S2 Excimer properties of 5 A** Monomer:excimer ratio varies linearly (r^2^ = 0.99) with proportion of HEPES **B** Absorbance is independent of HEPES content **C** Excitation scans for monomer (grey) and excimer (black) are essentially identical **D** Increasing the proportion of PBS increases emission from monomer and decreases emission from excimer **E** Monomer:excimer ratio varies linearly (r^2^ = 0.99) with proportion of PBS **F** Monomer intensity (relative to excimer) increases on decreasing concentration (performed in 20% HEPES / MeCN) **G** Similar results were obtained in MeOH. In all experiments [5] = 10 μM, λ_ex_ = 355 nm.


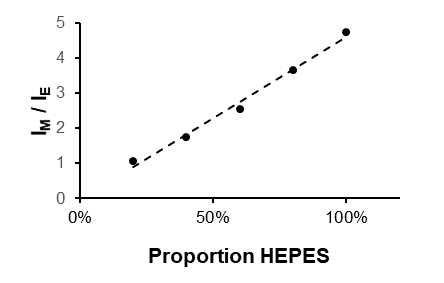


Increasing proportion PBS

Increasing proportion PBS


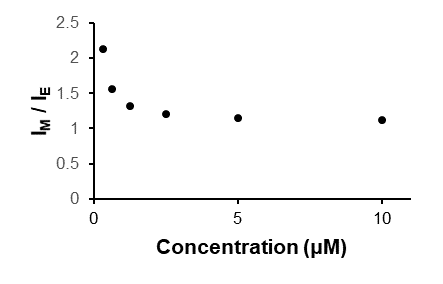


**A**

**D**

**G**


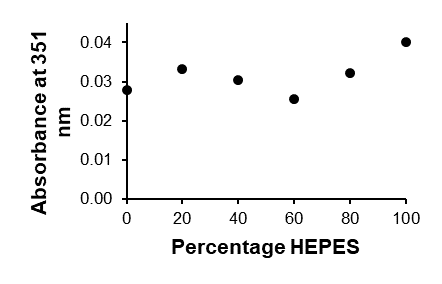

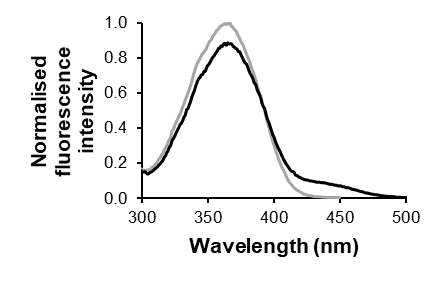


**E**

**F**

**C**

**B**

**Figure S3 Effect of adding Ca^2+^ on photophysical properties of 5 A** Fluorescence response is invariant with counterion (λ_ex_ = 355 nm) **B**, **C** Absorbance increases on addition of Ca^2+^. In all experiments [**5**] = 10 μM

**A**


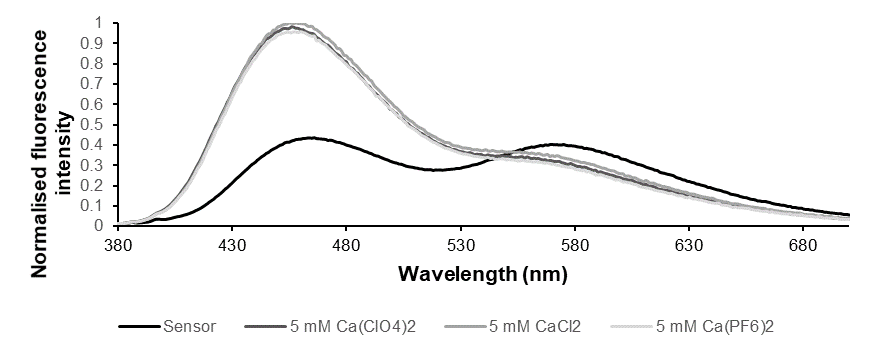

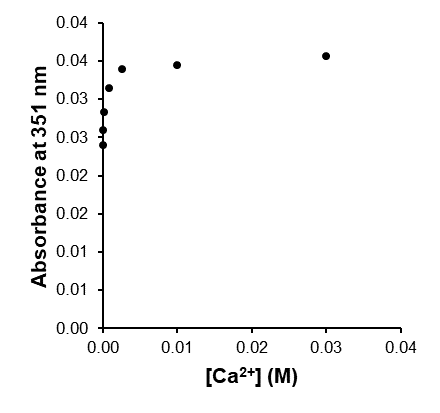

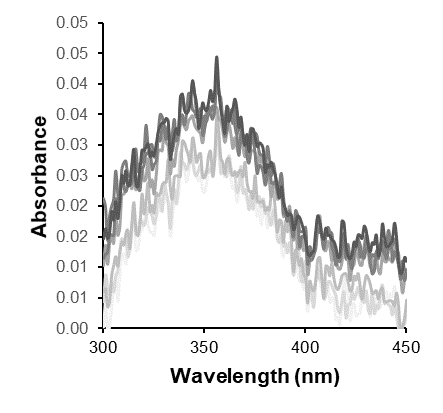


**B**

**C**

Increasing [Ca^2+^]


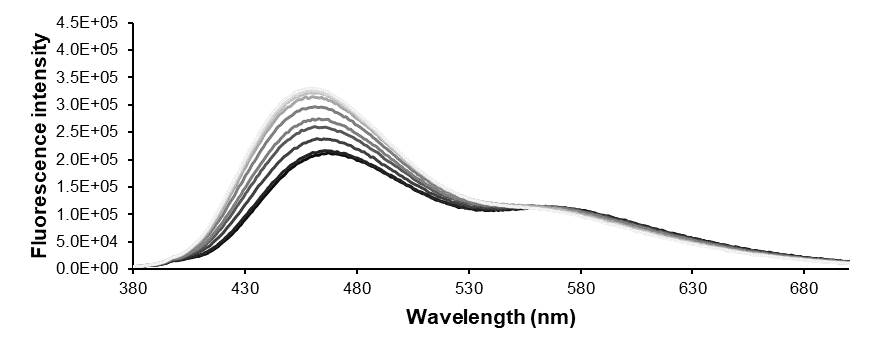

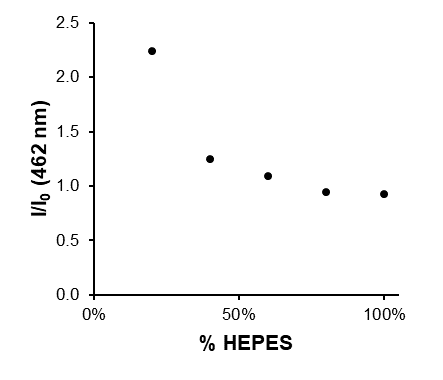


**Figure S4 Effect of varying proportions of aqueous on response of 5 to Ca^2+^ A** Titration experiment in 40% HEPES / MeCN; horizontal lines show approximate fluorescence intensity in the corresponding experiment using 20% HEPES. **B** Titration curve from experiment in in 40% HEPES / MeCN with a simple logarithmic fit. Curve fitting using BindFit and a 1:2 binding model yields a curve with lower residuals. **C** Fluorescence enhancement on addition of 5mM Ca^2+^ declines as proportion of HEPES increases. For all experiments [**5**] = 10 μM, λ_ex_ = 355 nm.

**B**

**C**

**A**


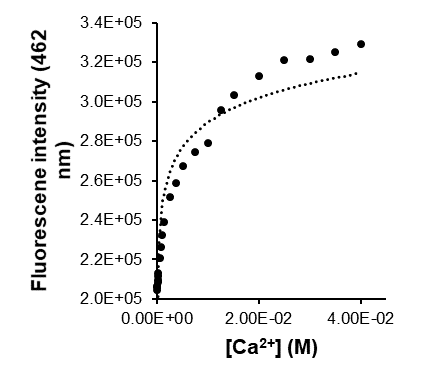


**A**

**B**

**Figure S5 Effect of cations on fluorescence of 5 A** Monovalent cations do not change fluorescence of 5 even at 100 mM (**B**) **C** Sensor 5 shows limited selectivity for Ca2+ over other group II metals **D** Some transition metal dications also enhance fluorescence **E** Other transition metal dications quench fluorescence **F** Cations often produce changes to the absorbance of **5**. (Cations were added as perchlorate salts as solutions in MeCN (with the exception of Me_4_NClO_4_ which was added as a solution in DMSO) to a final concentration of 1 mM unless otherwise stated.) All experiments were conducted in 20% HEPES / MeCN using [**5**] = 10 μM, λ_ex_ = 355 nm.


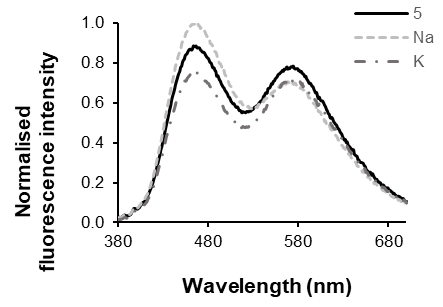

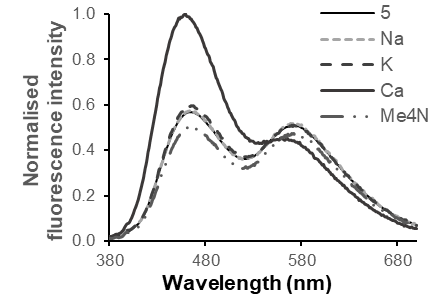

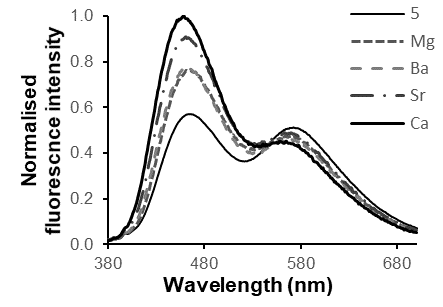

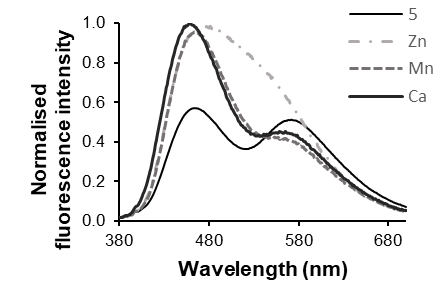

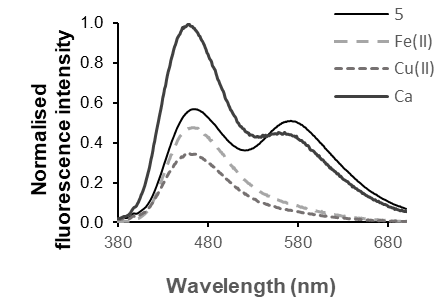

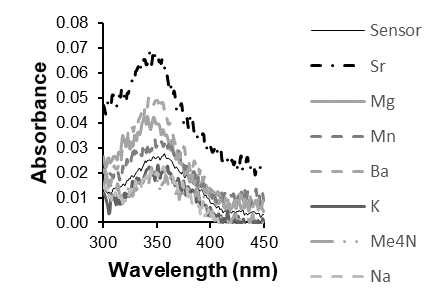


**F**

**C**

**D**

**E**


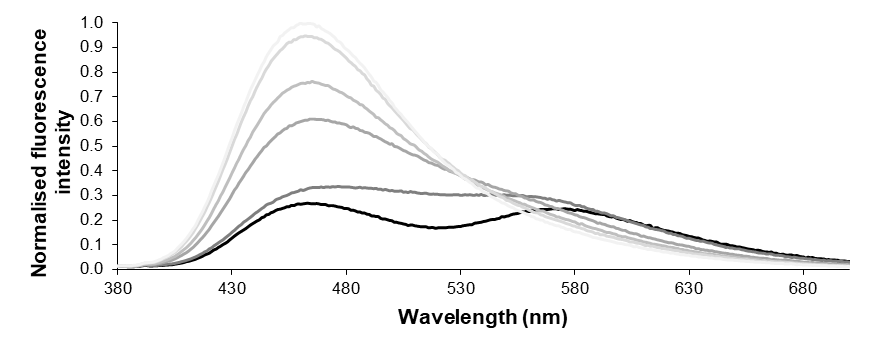

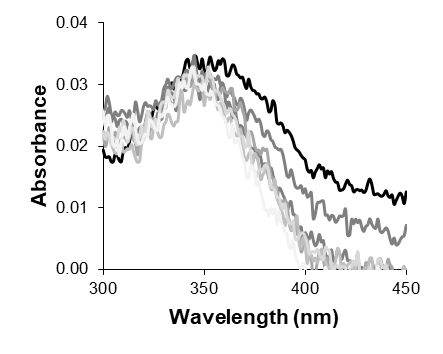


Increasing [Zn^2+^]

Increasing [Zn^2+^]

Increasing [Zn^2+^]

**A**

**B**

**C**


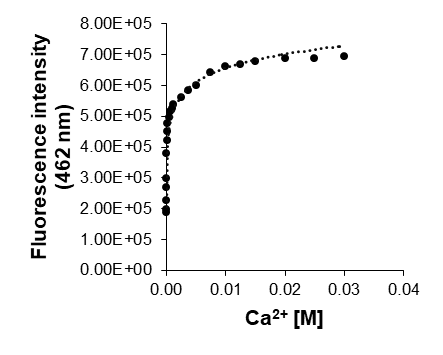


**Figure S6 Effect of addition of Zn^2+^ to 5 A** Titration experiment in 20% HEPES / MeCN **B** Titration curve (simple logarithmic fit) from A; a curve with lower residuals (obtained from BindFit) results from a 1:2 binding model and gives log*K_11_* = 4.0 **C** Absorption spectral changes on addition of Zn^2+^. For all experiments [**5**] = 10 μM, λ_ex_ = 355 nm.

**References**

(1) Hoang, M. D.; Bodin, J. B.; Savina, F.; Steinmetz, V.; Bignon, J.; Durand, P.; Clavier, G.; Méallet-Renault, R.; Chevalier, A. “CinNapht” Dyes: A New Cinnoline/Naphthalimide Fused Hybrid Fluorophore. Synthesis, Photo-Physical Study and Use for Bio-Imaging. *RSC Adv* **2021**, *11* (48), 30088–30092. https://doi.org/10.1039/d1ra05110e.

(2) Brynn Hibbert, D.; Thordarson, P. The Death of the Job Plot, Transparency, Open Science and Online Tools, Uncertainty Estimation Methods and Other Developments in Supramolecular Chemistry Data Analysis. *Chemical Communications* **2016**, *52* (87), 12792–12805. https://doi.org/10.1039/C6CC03888C.
